# Microbial Metabolites Determine Host Health and the Status of Some Diseases

**DOI:** 10.3390/ijms20215296

**Published:** 2019-10-24

**Authors:** Panida Sittipo, Jae-won Shim, Yun Kyung Lee

**Affiliations:** Department of Integrated Biomedical Science, Soonchunhyang Institute of Medi-Bio Science, Soonchunhyang University, Cheonan 31151, Korea

**Keywords:** intestinal microbiota, metabolite, intestinal epithelial cell, immune cell, inflammatory bowel disease, metabolic disease

## Abstract

The gastrointestinal (GI) tract is a highly complex organ composed of the intestinal epithelium layer, intestinal microbiota, and local immune system. Intestinal microbiota residing in the GI tract engages in a mutualistic relationship with the host. Different sections of the GI tract contain distinct proportions of the intestinal microbiota, resulting in the presence of unique bacterial products in each GI section. The intestinal microbiota converts ingested nutrients into metabolites that target either the intestinal microbiota population or host cells. Metabolites act as messengers of information between the intestinal microbiota and host cells. The intestinal microbiota composition and resulting metabolites thus impact host development, health, and pathogenesis. Many recent studies have focused on modulation of the gut microbiota and their metabolites to improve host health and prevent or treat diseases. In this review, we focus on the production of microbial metabolites, their biological impact on the intestinal microbiota composition and host cells, and the effect of microbial metabolites that contribute to improvements in inflammatory bowel diseases and metabolic diseases. Understanding the role of microbial metabolites in protection against disease might offer an intriguing approach to regulate disease.

## 1. Introduction

The gastrointestinal (GI) tract is a highly complex organ composed of the intestinal epithelium layer, microorganisms, and local immune system. In humans, microorganisms are most abundant within the intestinal tract [1,2,3], where various taxa including bacteria (>35,000 bacterial species), eukaryotes, viruses, and archaea reside [4,5]. Bacteria within the GI tract are called the ‘intestinal microbiota’. The intestinal microbiota is a microbial organ within the host as it is composed of different bacterial populations with the specific ability to communicate with each other and with the host cells [6]. The intestinal microbiota has co-evolved with the host, providing specific genetic and metabolic attributes. For example, the microbiota contains anaerobic bacteria that break down ingested polysaccharides, the most abundant biological polymer, supplying carbon and energy resources for its own growth and for the host [7]. Different sections of the GI tract contain distinct populations of the intestinal microbiota [8,9]. Pyrosequencing analysis of the intestinal microbiota composition in mice revealed that the phylogenetic diversity in the stomach and colon is higher than that in the small intestine, with Lactobacillaceae primarily found in the stomach and small intestine, and Bacteroidaceae, Prevotellaceae, Rikenellaceae, Lachnospiraceae, and Ruminococcaceae predominantly colonizing the colon [8]. Variation in intestinal microbial communities might be associated with differences in ingested foods and the host GI environment. Therefore, the intestinal microbiota is adapted to live in GIs that afford appropriate substrates for survival.

The intestinal microbiota converts ingested food or host products into metabolites, which target either the intestinal microbial population or host cells. Hence, the presence of metabolites depends on the microbial metabolic activity [10,11]. It is estimated that more than 50% of metabolites found in fecal matter and urine are derived from, or modified by, the intestinal microbiota [12]. According to a modified metabolite classification, the metabolites can be divided into three types as follows: (1) metabolites of dietary components that are transformed by the intestinal microbiota (e.g., compound K); [13] metabolites secreted by the host cell and modified by the intestinal microbiota (e.g., secondary bile acids); [14] metabolites that are synthesized by the intestinal microbiota de novo (e.g., short-chain fatty acids (SCFAs) [15,16]. Moreover, microbial metabolites are not only localized in the GI tract, but also broadly penetrate host tissue, primarily through the small intestine [17]. The effect of metabolites on the host cell, such as the promotion of intestinal epithelial regeneration and barrier integrity or the regulation of mucosal immune homeostasis, depends on the target cell type [18,19]. Therefore, the composition of the intestinal microbiota impacts host development, health, and disease.

Recently, many studies have focused on the modulation of intestinal microbiota and their metabolites for health improvement and disease prevention or treatment. Herein, we aim to provide an overview of up-to-date studies highlighting the potential implementation of metabolites for health and disease improvement. Further, we focus on the production of microbial metabolites and their biological impact on the intestinal microbiota composition and host cells, as well as the contribution of metabolites to the onset of inflammatory bowel diseases (IBDs), non-alcoholic fatty liver disease (NAFLD), obesity, and metabolic diseases.

## 2. Production of Microbial Metabolites and Their Effect on Microbial Clades

**Figure 1:**
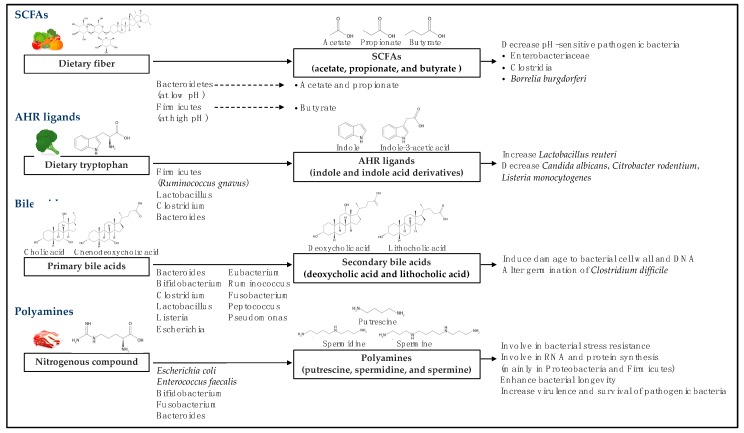
Conversion of dietary components and host-derived molecules to metabolites and their effect on microbial clades. Abbreviations: SCFAs, short-chain fatty acids; AHR, aryl hydrocarbon receptor.

The presence of intestinal microbiota can indicate specific microbial metabolites and the consequent effects on the bacterial microbiome and host cells [15,16]. In this section we discuss the biotransformation of bioactive metabolites, SCFAs, aryl hydrocarbon receptor ligands, bile acids, polyamines, and others, the metabolite-specific intestinal microbiota, and the effect of each metabolite on the intestinal microbiome (Figure 1).

### 2.1. Short-Chain Fatty Acids

SCFAs comprise the most thoroughly studied microbial metabolites. They are saturated aliphatic organic acids with a backbone of one to six carbons that can reach millimolar concentrations locally [20]. SCFAs are synthesized by the intestinal microbiota de novo [15,16]. Dietary fiber containing non-digestible carbohydrates, which escape digestion and absorption in the small intestine, is converted into SCFAs through intestinal microbial fermentation, primarily occurring in the colon [21]. Furthermore, non-digested proteins or peptides can also serve as substrates for SCFA production [22,23,24]. SFCAs can be further utilized for lipid or glucose synthesis. Hence, intestinal microbial-derived SCFAs provide an additional energy source to host cells such as colonocytes [25,26]. Acetate, propionate, and butyrate are the most abundant SCFAs in the GI tract (comprising ≥95% of total SCFAs), with a small proportion of SCFAs comprised of formate, valerate, and caproate [26]. The ratio of SCFAs in the colon is influenced by various factors, including the intestinal microbiota composition, the substrate, and host status [25]. The intestinal microbiota converts dietary fiber to SCFAs via three major metabolic pathways, including hydrolysis, glycolysis, and the pentose–phosphate pathway [26]. Acetate and propionate are primarily produced by Bacteroidetes, whereas butyrate is primarily generated by Firmicutes [11,27]. Acetate is mainly produced by *Lactobacillus* spp., *Bifidobacterium* spp., *Akkermansia muciniphila*, *Bacteroides* spp., *Prevotella* spp., *Ruminococcus* spp., and *Streptococcus* spp. via the Wood–Ljungdahl and acetyl-CoA pathways [27,28]. Propionate is produced by *Phascolarctobacterium* spp., *Bacteroides* spp., *Dialister* spp., *Veillonella* spp., *Salmonella* spp., *Roseburia inulinivorans, Ruminococcus obeum, Megasphaera elsdenii,* and *Coprococcus catus* via the succinate, acrylate, and propanediol pathways [27,29]. Butyrate is produced by *Roseburia* spp., *Clostridium leptum*, *Eubacterium hallii, Coprococcus eutactus, Faecalibacterium prausnitzii, Eubacterium rectale*, and *Anaerostipes caccae* via the butyryl-CoA:acetate CoA-transferase routes and the phosphotransbutyrylase/butyrate kinase pathway [27,29].

Microbial SCFA production results in reduced pH in the colon, which impacts the intestinal microbiota composition, including that of the dominant SCFA-producing bacteria and pH-sensitive pathogenic bacteria. For example, SCFAs reduce the growth of Enterobacteriaceae, including *Escherichia coli*, *Salmonella* spp., and *Clostridia* [30,31,32], in addition to inhibiting the metabolism and virulence of *Borrelia burgdorferi* [13]. Further, succinate and lactate, the byproducts of SCFA formation, are utilized by the intestinal microbiota for survival [33].

### 2.2. Aryl Hydrocarbon Receptor Ligands

AHR is a ligand-activated transcription factor that was recently highlighted as an important regulator of inflammation and immunity [34,35]. AHR ligands bind to AHR in various cell types, including immune cells, epithelial cells, and some tumor cells, and trigger subsequent effects [36]. Three sources of AHR ligands exist, specifically dietary, endogenous, and intestinal microbial-derived [36]. Many dietary components such as flavones, isoflavones, flavanones, and carotenoids are AHR agonists [37,38]. The majority of investigated dietary AHR ligands are generated from plant constituents such as glucobrassicin in cruciferous vegetables. The hydrolysis of glucobrassicin results in the formation of indole-3-carbinol (I3C) and indole-3-acetonitrile, both of which are AHR agonists [36]. Furthermore, dietary tryptophan (abundant in milk, eggs, red meat, and vegetables) is a major physiological reservoir for AHR ligand synthesis. Tryptophan is catabolized by intestinal microbiota to yield indole and indole derivatives [39]. The intestinal microbiota utilizes many pathways for tryptophan metabolism. For example, Firmicutes (*Clostridium sporogenes* and *Ruminococcus gnavus*) decarboxylate tryptophan to tryptamine, whereas Lactobacillus, Clostridium, and Bacteroides convert indole acetic acid from tryptophan to skatole [40]. Both tryptamine and skatole are AHR ligands. In addition, Lactobacillus utilizes tryptophan as an energy source and generates AHR ligands such as indole-3-aldehyde [41].

The presence of AHR ligands in the GI tract influences the intestinal microbial composition by inducing the expansion of *Lactobacillus reuteri* and inhibiting the growth of pathogenic bacteria [41,42,43]. Activation of AHR signaling in group 3 innate lymphoid cells (ILC3s) induces the production of interleukin (IL) 22, which drives the secretion of antimicrobial peptides. In this manner, AHR ligands can protect the host from pathogenic infection by *Candida albicans* [11,41]. AHR deficiency or lack of AHR ligands in mice results in perturbations in the intestinal microbial composition, causing the animal to become more susceptible to infection by *Citrobacter rodentium* and *Listeria monocytogenes* [42,43].

### 2.3. Bile Acids

Secondary bile acids are metabolites secreted by host cells and modified by the intestinal microbiota. In humans, a sequence of enzymatic reactions (involving more than 17 enzymes) in the liver converts cholesterol to primary bile acids (cholic and chenodeoxycholic acids), which can be further converted to secondary bile acid metabolites by the intestinal microbiota [44,45]. The cholesterol-derived primary bile acids are either returned to the liver (enterohepatic circulation) or travel to the colon where they are then transformed through bacterial metabolism. Colonic microbiota converts the primary bile acids to secondary bile acids via various reactions, including deconjugation, oxidation and epimerization, dehydroxylation, esterification, and desulfatation, resulting in the formation of 16 different bile acids in early life and more than 20 bile acids in adult humans [44,45,46]. Deconjugation is driven by bile salt hydrolases, which have been identified in *Bacteroides fragilis*, *Bacteroides vulgatus*, *Listeria monocytogenes*, Clostridium, Lactobacillus, and Bifidobacterium. Oxidation and epimerization require bile acid hydroxysteroid dehydrogenases produced by intestinal Firmicutes (including Clostridium, Eubacterium, and Ruminococcus), Bacteroides, and Escherichia. Dehydroxylation only occurs after deconjugation, and is catalyzed by members of the Firmicutes phylum, including Clostridium (*C. scindens* or *C. hylemonae*) and Eubacterium. Esterification activity has been identified in few intestinal microbes, including Bacteroides, Eubacterium, and Lactobacillus. Desulfatation, driven by bile acid sulfatase, is catalyzed by Clostridium, Peptococcus, Fusobacterium, and Pseudomonas [44,47]. The products of these processes are deoxycholic acid (DCA) and lithocholic acid (LCA). Most DCA is reabsorbed in the intestine or transported back to the liver, whereas LCA is primarily excreted in feces [48].

To date, the effect of bile acid metabolites on the intestinal microbiota has not been well studied. Some studies have demonstrated that bile acids can induce bacterial DNA and membrane damage; however, bacteria possess protective and repair mechanisms against bile acid-induced damage [49,50,51]. Nevertheless, the absence of intestinal bile acids can promote bacterial translocation from the intestine to visceral tissues [52]. In addition, the different forms of bile acids influence the initiation of *Clostridium difficile* spore germination [53,54]. For example, the production of LCA by *C. scindens* inhibits the germination of *C. difficile* [54].

### 2.4. Polyamines

Polyamines are small polycationic molecules that are derived from food or biosynthesized by the intestinal microbiota [55]. Polyamines such as putrescine, spermidine, and spermine are integral to a broad range of biological process. Nearly all food products contain polyamines, including soybean, green tea leaves, wheat germ, mushrooms, oranges, and meat. Most food-derived polyamines are absorbed in the small intestine [56]. However, high amounts of polyamines, especially putrescine and spermidine, in the colon are primarily produced by the intestinal microbiota, including *Bacteroides* spp. and *Fusobacterium* spp. [57,58,59]. Two main species of intestinal microbes, *E. coli* and *Enterococcus faecalis*, can biosynthesize putrescine from arginine using a hybrid pathway [60,61]. Arginine is converted to agmatine by *E. coli* [60] and then metabolized to putrescine by *E. faecalis* [61]. Further, the presence of *Bifidobacterium* spp. that produce acidic compounds accelerates the production of putrescine [62]. The production of spermidine is mediated by at least two types of bacteria, *E. coli* and intestinal bacteria from the genus Bacteroides. These bacteria utilize carboxyspermidine decarboxylase and spermidine synthase for spermidine biosynthesis [63,64].

Polyamines are crucial for a variety of biological mechanisms in the intestinal microbiota (primarily Proteobacteria and Firmicutes), including cellular signaling, stress resistance, and RNA and protein synthesis [65,66]. In addition, polyamines enhance bacterial longevity by promoting autophagy [67]. Several pathogenic bacteria depend on polyamines for their virulence and survival within the host, such as *Salmonella enterica* serovar Typhimurium, *Streptococcus pneumoniae*, and *Shigella* spp. [68,69]. Further, polyamines are required for biofilm formation by *Vibrio cholerae* and *Yersinia pestis* [70,71].

### 2.5. Others

#### 2.5.1. Equol

Equol is metabolized from isoflavone daidzein, which is commonly found in soybeans. Germ-free animals and newborn infants that lack a developed intestinal microbiota cannot form equol, suggesting that intestinal microbiota is involved in its formation [72,73]. In humans, many intestinal microbiota are responsible for the production of equol, such as Bifidobacterium, *Enterococcus faecium*, Lactobacillus, Ruminococcus, and Streptococcus [74]. β-glucosidases produced from brush border membrane and intestinal microbiota are necessary for deglycosylation of soy isoflavones (daidzin) to release bioavailable daidzein, which can be absorbed across enterocytes or further converted to equol [75,76]. Equol can inhibit the growth and spore formation of *Clostridium difficile,* suggesting that it has antimicrobial activity [77]. Moreover, equol also modulates the growth rate of certain intestinal microbiota such as *Bacteroides fragilis* and *F. prausnitzii* [78].

#### 2.5.2. Compound K

Compound K is a metabolite formed upon the transformation of dietary components by intestinal microbiota [15,16]. It is a microbial metabolite of ginsenoside Rb1, a 20(*S*)-protopanaxadiol-type ginsenoside and a component of ginseng [79,80,81]. Ginsenoside Rb1 has low membrane permeability and is easily degraded [82]. Hence, its biological effects are enhanced by intestinal microbial metabolism [80,83]. Ginsenoside Rb1 is metabolized through deglycosylation and hydrolysis by intestinal microbes such as Lactobacillus, Bifidobacterium, *Bacteroides thetaiotaomicron*, and *Streptococcus thermophilus* [80,81]. Non-digestible food ingredients and prebiotics stimulate the formation and absorption of compound K by increasing glycosidase activity and the growth of compound K-forming intestinal microbes such as Lactobacillus and Bacteroides [84,85]. Further, compound K can alter the composition of the intestinal microbiota by increasing the growth of bacteria from the genera Lactococcus and Clostridium, known producers of SCFAs [79]. Since the intestinal microbiota is involved in the production of multiple effective metabolites, modulation of the intestinal microbiota composition might be an alternative approach to regulate microbial metabolite levels, which could further determine the health and disease status of the host.

## 3. Microbial Metabolites: Messages from the Intestinal Microbiota to the Host Cell

The GI tract contains high levels of multiple microbial metabolites, which can interact with the host cells and, in particular, cells localized to the intestinal tract, including intestinal epithelial cells (IECs) and local immune cells [11,86,87]. However, microbial metabolites are not solely accumulated in the GI tract, but also penetrate host tissues, thereby triggering host metabolic and immunological responses [17]. In the section that follows, we provide an overview of how microbial metabolites influence host cells, specifically, IECs and immune cells (as summarized in Table 1 and Table 2, respectively).

### 3.1. Effect of SCFAs on Host Cells

#### 3.1.1. Effect on Intestinal Epithelial Cells (IECs)

**Figure 2:**
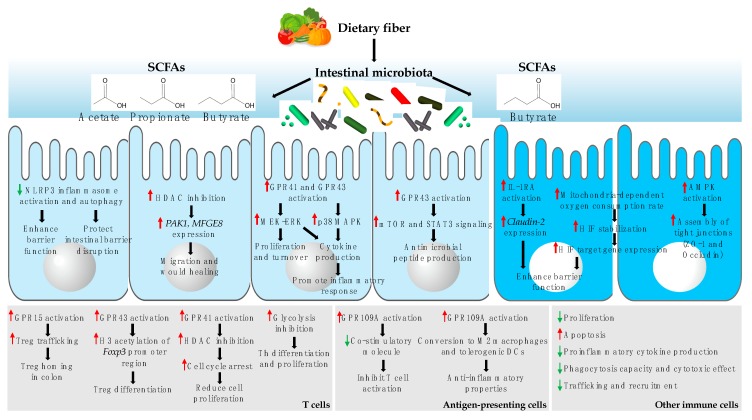
Dietary fiber-derived short-chain fatty acids (SCFAs) affect intestinal epithelial cells (IECs) and immune cells. SCFAs derived from dietary fiber impact IECs through various mechanisms. These effects include enhancing barrier functions, maintaining intestinal barrier integrity, inducing IEC migration and wound healing, promoting proliferation, turnover, and inflammatory responses, and inducing antimicrobial peptide production and the assembly of tight junction proteins. In addition, SCFAs affect immune cells, including T cells, antigen-presenting cells, and others such as monocytes and macrophages. SCFAs induce the differentiation and tissue homing of regulatory T cells (Tregs), promote the cell proliferation and differentiation of helper T (Th) cells, modulate the function of antigen-presenting cells, and alter other immune cell properties. Abbreviations: NLRP3, NOD-like receptor pyrin domain-containing protein 3; HDAC, histone deacetylase; PAK1, p21 activated kinase; MFGE8, milk fat globule-EGF factor 8; GPR, G-protein coupled receptor; MAPK, mitogen-activated protein kinase; Treg, regulatory T cell; Th, helper T cell; DCs, dendritic cells.

SCFAs, particularly butyrate, are a major energy source for IECs in the colon and reinforce the intestinal barrier function via multiple mechanisms [88,89,90,91]. SCFAs act as histone deacetylase (HDAC) inhibitors, which inhibit lipopolysaccharide (LPS)-induced activation of the NLRP3 inflammasome and autophagy, leading to enhanced intestinal barrier function and protection against intestinal barrier disruption [88]. According to a recent study, SCFA-induced HDAC inhibition also promotes wound healing by stimulating IEC migration through p21 activated kinase 1 (PAKI) and milk fat globule-EGF factor 8 (MFGE8) [86]. Butyrate induces the expression and activation of IL-10 receptor α-subunit and represses the expression of permeability-promoting claudin-2, which enhances the intestinal epithelial barrier function [89]. Butyrate also accelerates the mitochondrial oxygen consumption rate of IECs and stabilizes hypoxia-inducible factor and its target genes, improving the barrier function [90]. Further, butyrate accelerates the assembly of tight junction proteins, namely ZO-1 and occludin, by activating AMP-activated protein kinase [91]. SCFAs are also involved in IEC proliferation and function by activating G-protein coupled receptors (GPRs) [87,92,93]. For example, reduced IEC proliferation and turnover are restored when germ-free mice or antibiotic-treated mice are administered a mixture of SCFAs; this effect could be mediated by the activation of MEK–ERK signaling through GPR41 or GPR43 [92]. Indeed, SCFA-induced activation of these GPRs in IECs triggers the MEK–ERK and p38 MAPK pathways, leading to the production of cytokines by IECs [93]. Further, SCFA-induced GPR43 activation promotes the production of antimicrobial peptides like RegIIIϒ and β-defensin by IECs by activating mTOR and STAT3 signaling [87]. Butyrate also induces IEC differentiation and enhances apoptosis; however, the detailed mechanisms involved have not yet been elucidated [94]. Finally, acetate and butyrate increase the secretion of glucagon-like peptide-1 (GLP-1) by L cells [95]. The effects of SCFAs on IECs are summarized in Figure 2.

#### 3.1.2. Effect on Immune Cells

The effect of SCFAs on the immune system, particularly the anti-inflammatory effect, has been shown by many studies [11,19]. SCFAs are physiological ligands for different GPRs, including GPR109A, GPR41, and GPR43, which are differentially expressed by various immune cell types, activating signaling cascades related to immune cell maturation and function [11,96,97,98]. Butyrate is a ligand of GPR109A that is expressed on macrophages, dendritic cells (DCs), neutrophils, ILCs, and T cells [99,100,101]. Propionate is a ligand of GPR41 and GPR43, whereas acetate is a ligand of GPR43; these two GPRs are expressed on monocytes, DCs, eosinophils, neutrophils, and T cells [102,103]. The activation of GPRs sequentially leads to a decrease in the production of pro-inflammatory mediators through different mechanisms. Butyrate inhibits HDAC and nuclear receptor peroxisome proliferator-activated receptor gamma, resulting in the reduction of pro-inflammatory cytokine expression mediated by nuclear factor kappa-light-chain-enhancer of activated B cells (NF-κB) [104,105,106]. In addition, SCFAs, and primarily butyrate, modulate the recruitment of immune cells to the intestinal tract, by controlling the expression of chemokines or chemoattractant receptors; for example, propionate and butyrate suppress leukocyte trafficking by reducing the production of several chemokines, including CCL3, CCL4, CCL5, CXCL9, CXCL10, and CXCL11, by human monocyte-derived DCs [97]. Acetate acts on GPR43 to decrease the expression of chemoattractant receptors, including C5aR and CXCR2, in neutrophils, resulting in the reduction of neutrophil recruitment to the intestinal tract [103]. SCFAs also promote the GPR15-dependent homing of Tregs in the colon [107]. Further, butyrate affects T cell proliferation in a concentration-dependent manner. T cell proliferation is stimulated by low butyrate levels (and inhibited by high butyrate levels), which might be related to alterations in cell cycle progression. This effect might be mediated by the interaction between butyrate and GPR41, followed by HDAC inhibition [108,109]. In addition, HDAC inhibition by butyrate induces the apoptosis of immune cells such as macrophages, neutrophils, mast cells, and T cells [109,110,111]. Furthermore, butyrate controls immune responses by modifying the function of antigen-presenting cells, such as reducing the expression of co-stimulatory molecules and inducing the anti-inflammatory properties of DCs and macrophages, which is mediated by GPR109A activation. This results in reduced pro-inflammatory cytokine production and Treg differentiation [112]. Lastly, SCFAs contribute to Treg differentiation by increasing histone H3 acetylation in the *Foxp3* promoter region, which is mediated by GPR43 activation [113,114]. The overall effects of SCFAs on immune cells are summarized in Figure 2.

### 3.2. Effect of AHR Ligands on Host Cells

#### 3.2.1. Effect on IECs

The AHR ligand indole increases the expression of genes involved in tight junction formation, mucin production, and the attenuation of NF-κB activation, decreasing the production of pro-inflammatory cytokines (such as IL-8), while increasing the production of anti-inflammatory cytokines (such as IL-10) [115]. The administration of indole also significantly upregulates IL-10 receptor levels in IECs [116]. As shown in intestinal organoid culture, I3C affects IEC differentiation by suppressing enterocyte differentiation (by reducing the expression of intestinal alkaline phosphatase) and inducing the differentiation of secretory cells such as Paneth and goblet cells (by increasing lysozyme and mucin-2 expression, respectively) [117]. This mechanism is mediated by alterations in Wnt and Notch signaling.

#### 3.2.2. Effect on Immune Cells

AHR ligands such as tryptamine and indole-3-acetate attenuate the inflammatory responses of macrophages and inhibit macrophage migration toward the chemokine MCP-1 [10]. AHR ligands suppress the production of pro-inflammatory cytokines such as IL-1β and IL-6 by macrophages through several mechanisms [118,119]. To control IL-6 expression, AHR forms a complex with STAT1 and NF-κB, which inhibits *Il6* promoter activity [118]. Further, the interaction between AHR and SP1 represses the expression of histidine decarboxylase, resulting in decreased histamine production, which further reduces the activation of histamine receptor-dependent IL-6 production in macrophages [119]. Ligand-bound AHR activates the expression of an inhibitor of caspase-1 activation, PAI-2, resulting in reduced IL-1β secretion [120]. In addition, AHR ligands induce bacterial clearance by inducing the apoptosis inhibitor AIM, promoting macrophage survival and increasing the production of reactive oxygen species (ROS) by macrophages [121]. AHR activation is also required for ILC3 expansion and the production of IL-22 [122]. AHR ligands are critical regulators of T cell differentiation; for example, activated AHR binds STAT1 and STAT5, which inhibits Th17 development [123]. In addition, indole regulates Treg/Th17 lineage fate by inducing the expansion, function, and stability of Tregs, while suppressing STAT3 and RORγt-meditated Th17 development [124]. Furthermore, AHR ligands such as I3C are necessary for the maintenance of intraepithelial lymphocytes and are essential for immune functions [125].

### 3.3. Effect of Bile Acids on Host Cells

#### 3.3.1. Effect on IECs

Bile acids produced by the intestinal microbiota are considered essential to solubilize dietary fat and cholesterol, contributing to their digestion and absorption, particularly in the small intestine [96]. They act as signaling molecules by activating the nuclear receptor farnesoid X receptor (FXR) or the plasma membrane receptor G-protein–coupled receptor 5 (TGR5), which are expressed on IECs in the small intestine and colon [44,126]; for example, microbial bile acid derivatives DCA and LCA bind to FXR and regulate IEC integrity [127]. In addition, the interaction between DCA (at physiological concentrations) and FXR can inhibit the EGFR/Src/ERK pathway in IECs, resulting in reduced IEC proliferation [128]. However, bile acids can also stimulate the proliferation and invasiveness of colon cancer cells via the AP1 and c-Myc pathways [129]. In addition, the colonization of *C. scindens*, which mediates primary-to-secondary bile acid conversion, induces liver tumor growth by inhibiting the production of chemokine CXCL16 by liver sinusoidal endothelial cells and reducing the accumulation of natural killer T cells in the liver [130]. One IEC lineage, enteroendocrine L-cells, also senses bile acids through FXR and TGR5, which regulates GLP-1 secretion. After the activation of TGR5 by bile acids, the levels of intracellular cAMP and calcium increase, consequently enhancing GLP-1 secretion [131]. In addition, deconjugated forms of bile acids might increase the turnover of colonocytes [132].

#### 3.3.2. Effect on Immune Cells

Bile acid receptors TGR5 and FXR are also expressed by several immune cells. However, microbial bile acid derivatives DCA and LCA are preferential agonists of TGR5. A recent transcriptome analysis revealed that bile acids affect cytokine production patterns, reducing pro-inflammatory cytokine and chemokine production, while inducing anti-inflammatory effects and wound healing and reprograming pro-inflammatory macrophages into anti-inflammatory cells [133]. An investigation of bone marrow-derived macrophages (BMDMs) demonstrated that LCA is a potent activator of the NLRP3 inflammasome, and LCA treatment was found to block caspase-1 maturation and IL-1β and IL-18 secretion by LPS-primed BMDMs via the TGR5–cAMP–PKA axis [134]. Indeed, TGR5 signaling activated by bile acids in turn activates the cAMP–PKA pathway, leading to the phosphorylation and ubiquitination of the NLRP3 inflammasome and the inhibition of caspase-1-dependent maturation and the release of pro-inflammatory cytokines (IL-1β and IL-18). Interactions between LCA and vitamin D receptor on both human and mouse T cells decrease ERK1/2 phosphorylation, inhibiting Th1 activation, as determined by the decreased production of Th1-associated cytokines and decreased STAT1 phosphorylation [135].

### 3.4. Effect of Polyamines on Host Cells

#### 3.4.1. Effect on IECs

Polyamines, and mainly putrescine, increase DNA synthesis in IECs. This effect might be mediated by the modulation of enzymatic activity that is integral to the initiation of DNA synthesis or stimulation of the initiation factors [136]. IEC migration from the crypts to the villi is essential for the organization and integrity of the intestinal mucosa and for the repair or healing of the damaged intestinal mucosa. Polyamines induce the activation of Rac1 and sequentially activate RhoA and Cdc42, which are important for actin polymerization, resulting in IEC migration [137]. It has been shown that polyamine-activated Rac1 forms a complex with PLCγ1, increasing calcium influx and promoting cell migration [138]. Polyamines might also be involved in intestinal maturation; for example, spermidine and spermine induce galactosyltransferase activity and glycoprotein galactosylation, which are important for the biosynthesis of mucins and most enzymes in the IEC brush border membrane [139]. Furthermore, spermine might suppress the production of IL-18 and antimicrobial peptides by IECs resulting from a reduction in NLRP6 inflammasome assembly [140].

#### 3.4.2. Effect on Immune Cells

Polyamines exert an anti-inflammatory effect. The uptake of spermine by monocytes is enhanced by LPS stimulation, and the inhibition of monocyte polyamine uptake restores monocyte TNF synthesis [141]. Therefore, spermine might inhibit monocyte activation and pro-inflammatory cytokine production. In addition, in cultured LPS-induced human peripheral blood mononuclear cells, IL-1, MIP-1α, and MIP-1β synthesis is inhibited by the presence of spermine [142]. Further, the daily administration of either spermine or spermidine to rats from birth affects intestinal and systemic immune cell maturation [143]. Specifically, spermine and spermidine accelerate the maturation of CD8^+^ T cells, enhance the presence of intraepithelial natural killer cells, and elevate the percentage of mature CD4^+^ lymphocytes in the lamina propria, in addition to promoting early B cell maturation (inducing an increase in the percentage of IgM^+^ cells). Even though polyamines have been linked to immune functions, little is known about the mechanism whereby they exert biological functions.

### 3.5. Effect of Other Metabolites on Host Cells

#### 3.5.1. Effect on IECs

Equol can protect IECs from oxidative damage by promoting the expression of antioxidant genes, enhancing antioxidant enzyme activity [144]. In addition, equol can also maintain the integrity of the tight junctions and inhibit IL-8 production by IECs [145]. Enteroendocrine L cells express putative equol receptors, GPR30 and estrogen receptors. The interaction between equol and receptors leads to increased levels of intracellular Ca^2+^ and actin reorganization, resulting in the suppression of GLP-1 secretion [146]. An in vitro study with Caco-2 cells showed that the ginsenoside metabolite compound K induces glucose uptake mediated by the Na(+)/glucose co-transporter 1 (SGLT1), which is essential to ameliorate intestinal inflammation. Compound K induces EGFR phosphorylation, which is functionally required for CREB and CBP binding to the *SGLT1* promoter, changing the *SGLT1* chromatin to an activated status, resulting in glucose uptake by IECs [147]. Compound K also inhibits IL-8 secretion by LPS-activated human colorectal cancer cell lines, suggesting an anti-inflammatory effect [148]. In addition, compound K can arrest the cell cycle in the G1 phase, resulting in the inhibition of cell growth and the induction of ROS generation, leading to cell apoptosis by modulating the mitochondrion-dependent apoptotic and MAPK pathways [148,149].

#### 3.5.2. Effect on Immune Cells

Equol decreases the production of IL-12/IL-18-induced interferon-gamma (IFN-γ) production by natural killer cells [150]. Equol can protect macrophages from LPS-induced oxidative stress by reducing lipid peroxidation products and enhancing the activity of antioxidant enzymes [151]. In addition, mice administered equol showed higher antigen-specific IgE production from B cells and IL-13 production from T cells compared to those in the control group [152]. Compound K suppresses the production of pro-inflammatory cytokines [153,154]. Treatment of LPS-stimulated murine peritoneal macrophages with compound K inhibits the activation of IL-1 receptor associated kinase-1, leading to NF-κB inactivation and reduced pro-inflammatory cytokine production [153]. Compound K also affects macrophage function, including polarization and endocytosis by inhibiting the expression of β-arrestin 2, a negative regulator and scaffolding protein of GPRs. Further, compound K inhibits the formation of M1 macrophages, as indicated by the amelioration of inflammatory responses and reductions in pro-inflammatory cytokine (IL-1β, TNF-α, and IL-17) secretion [154]. Similar to the effect on IECs, compound K also induces cell cycle arrest in G1 phase and apoptosis in U937 human monocytic leukemia cells, which is mediated by upregulated p21 expression and elevated JNK activation [155]. In addition, compound K suppresses the CCL2/CCR-mediated migration of DCs and can also inhibit signals for T cell activation, including major histocompatibility complex class II and co-stimulatory molecules such as CD80 and CD86, resulting in the suppression of T cell priming and the reduction of a population of activated T cells [156]. Finally, compound K can be cytotoxic, as it reduces T cell viability under Treg differentiation conditions in vitro [157].

These observations have led to the elucidation of specific metabolite functions and the underlying mechanisms. Collectively, most metabolites exert an anti-inflammatory effect necessary for the maintenance of tissue homeostasis. However, high levels of metabolites might generate negative feedback in the host; for example, prolonged anti-inflammatory effects might increase the risk of infectious disease. Therefore, maintaining a balanced intestinal microbiota and microbial-derived metabolites is important for host health. Importantly, this microbiota–host relationship is mutualistic in that it is not only the microbial metabolites that impact host cells, but also the host that influences the presence of microbial metabolites. Specifically, the host immune system affects the presence of microbial metabolites; for example, secretory antibodies can reduce the time that microbiota reside in the small intestine and limit the penetrative capacity of microbial metabolites to other tissues [17]. Hence, alterations to the host immune system might induce an imbalance in the microbiota, thereby altering the release of microbial metabolites. Imbalanced intestinal microbiota or the lack of representative metabolites can lead to host diseases, as discussed in the next section.

## 4. Metabolites and Diseases

Based on the activity of microbial metabolites described previously herein, these molecules are considered key metabolic regulators capable of influencing different organs in the body. The contribution of metabolites to the onset of IBD, NAFLD, obesity, and metabolic diseases, with a particular focus on the association with type 2 diabetes (T2D) and cardiovascular disease (CVD), has been extensively documented.

### 4.1. IBDs

IBDs are a group of multifactorial chronic inflammatory diseases of the GI tract. Crohn’s disease and ulcerative colitis are the most common forms of IBD. Many studies have documented a significant shift in intestinal microbiota composition in IBD patients, indicating that changes in the intestinal microbiota, as well as the associated metabolites, might be related to these diseases [158,159]. Moreover, changes in microbial metabolites might contribute to impaired interactions between metabolites and host cells, notably in IECs and immune cells, as discussed. Most bacterial metabolites play an immunomodulatory role [41,118,119,125]; for example, in a mouse model, colitis development is exacerbated by changes in the intestinal microbiota composition caused by the absence of AHR or its ligands [125]. As mentioned, Firmicutes, Lactobacillus, Clostridium, and Bacteroides utilize dietary tryptophan as an energy source and produce the AHR ligand indole-3-aldehyde. This metabolite activates AHR-expressing ILC3s, leading to cell expansion and the production of IL-22, driving the secretion of antimicrobial peptides and promoting mucosal healing [41,122]. In addition, the loss of AHR activation might lead to the disruption of Treg/Th17 cell populations by reducing Treg stability and inducing the development of Th17 cells, as observed in patients with IBDs [124,160]. Furthermore, impaired AHR activation in IECs might interfere with anti-inflammatory cytokine production and tight junction formation, contributing to IBD development [115]. A reduction in SCFA-producing microbial species is also associated with IBDs [161]. This might be explained by the HDAC-inhibiting activity of SCFAs, particularly butyrate, which results in a reduction in NF-κB-mediated cytokine production by immune cells, indicating a protective effect of SCFAs on IBDs [104,105,106]. In support of this, butyrate was reported to protect mice from IBD by inducing the expression and activation of IL-10 receptor α-subunit, which results in increased anti-inflammatory activity, enhanced epithelial barrier function, and wound healing [86,89]. Further, compound K promotes recovery from colitis in a mouse model by inhibiting the NF-κB-mediated inflammatory response [162]. However, many microbial metabolites associated with IBDs remain to be identified.

### 4.2. NAFLD

NAFLD has become the most common chronic liver disease with an ever increasing incidence worldwide [163]. The pathogenesis of NAFLD is defined by abnormal levels of fat in hepatocytes, inflammation, and cell damage [164]. Microbial metabolites have been described as being associated with NAFLD pathogenesis. For example, SCFAs have a beneficial role in hepatic metabolism and function [165,166,167]. Further, acetate supplementation reduces hepatic fat accumulation and decreases hepatic inflammation [165,166]; further, butyrate enhances mitochondrial function by improving respiratory capacity and fatty acid oxidation [167]. LPS also plays an important role in the progression of NAFLD [168], whereas butyrate has a major role in maintaining intestinal integrity by enhancing the expression of tight junctions [89,91]. Therefore, butyrate might prevent the transport of LPS to the liver, thereby indirectly suppressing the progression of NAFLD. Both acetate and butyrate induced the secretion of GLP-1, which can prevent NAFLD by increasing fatty acid oxidation, decreasing lipogenesis, and improving hepatic glucose metabolism [95,169]. Moreover, bile acids bind the nuclear receptor FXR, which is expressed in many cell types, including hepatocytes. The activation of FXR has beneficial effects on hepatic lipid and carbohydrate metabolism, which can suppress NAFLD [170]. Lastly, the AHR ligands tryptamine and I3A have also been shown to alleviate NAFLD by reducing cytokine-mediated lipogenesis in hepatocytes [10]. However, a translational study examining the beneficial effect of microbial metabolites on NAFLD is required.

### 4.3. Obesity

Obesity can result when energy intake exceeds energy expenditure, leading to the excessive accumulation of fat in adipocytes and the development of adipose tissue inflammation accompanied by an increase in the production and secretion of pro-inflammatory adipokines [171]. Numerous studies have shown that SCFAs are associated with obesity by regulating appetite, energy intake, energy expenditure, and energy harvesting [172]. In a rodent study, SCFAs were found to stimulate the secretion of GLP-1 and peptide YY (PYY) via enteroendocrine cells [95]. Moreover, a study on human obese patients reported that oral administration of propionate increases plasma concentrations of GLP-1 and PYY, which is associated with an observed reduction in food intake [173]. Similarly, in obese mice, butyrate administration was found to result in decreased body weight driven by energy expenditure and lipid oxidation [174]. Further, another AHR ligand, indole, can modulate GLP-1 release from L-cells, which might contribute to the prevention of obesity [175]. The daily administration of polyamines, either spermidine or spermine, can effectively induce weight loss and improve insulin sensitivity in a diet-induced obesity mouse model [176]. Obesity is strongly linked to other diseases such as NAFLD and metabolic diseases; therefore, understanding how microbial metabolites affect obesity is critical.

### 4.4. Metabolic Diseases

As follows, we focus on T2D and CVD, two major metabolic diseases associated with alterations in intestinal microbiota and microbial metabolites. T2D is characterized by hyperglycemia caused by impaired insulin production by pancreatic beta cells and insulin-mediated suppression of glucose production in the liver. Differences in the intestinal microbiota composition were observed between healthy individuals and those with T2D, which might contribute to the onset of impaired insulin secretion [177]. However, GLP-1 produced by IECs can restore insulin and glucagon imbalances by activating the GLP-1 receptor on pancreatic beta cells. Further, many lines of evidence show that the colonization of SCFA-producing bacteria and the presence of SCFAs potentially improve diabetes, particularly T2D [178,179]. As mentioned in the previous section, SCFAs induce the secretion of GLP-1 by L cells and, therefore, SCFAs improve T2D by promoting insulin production and sequentially regulating glucose metabolism [95,178]. In addition to SCFAs, bile acids also induce the production of GLP-1, which was recently recognized as a new target for T2D treatment [131,180]. In addition, the levels of indolepropionic acid, a microbial tryptophan metabolite, are inversely associated with the incidence of T2D and positively associated with insulin secretion, which indicates that this metabolite might exert a positive effect on T2D by promoting insulin secretion by pancreatic beta cells [181]. Currently, dietary trends have shifted toward the increased consumption of non-fiber foods, resulting in excessive weight gain and insufficient supplies of substrates to generate microbial metabolites. Moreover, overweight or obese individuals are often at an increased risk of developing T2D.

CVD is a term encompassing disorders of the heart and blood vessels, which can cause stroke and heart attack. Obesity, a common feature of T2D, is highly associated with an increased risk of CVD [182]. The association between T2D and CVD might be explained by low-grade inflammation and lipid accumulation in adipose tissue, as well as increased levels of serum C-reactive proteins, which could lead to the development of endothelial dysfunction [182,183]. Increased consumption of dietary fiber is strongly associated with reduced C-reactive protein levels, implying that SCFAs derived from dietary fiber might reduce the risk of CVD [14]. In addition, supplementation with acetate (the primary energy source for the brain, liver, and muscles) improves cardiovascular function and prevents the development of CVD [29,184]. Since central nervous system (CNS) disorders can cause CVD [185], the manipulation of intestinal microbiota with psychobiotics (a group of probiotics that affect the CNS) might reduce the risk of CVD. Furthermore, according to an epidemiological study investigating the contribution of polyamines to CVD prevention, the consumption of a polyamine-containing diet is negatively associated with CVD risk [186]. Polyamines might improve CVD by suppressing pro-inflammatory cytokine production and improving endothelial cell function [142,187]. Furthermore, other microbial metabolites, including bile acids and compound K, might also reduce CVD risk by modulating cardiovascular function [188,189].

Dietary consumption behavior can influence the presence of intestinal microbiota and associated metabolites, which help to control host diseases [177,190]. For example, the consumption of sweetener decreases SCFA production and alters the colonic pH, which might increase the risk of diseases [191]. Moreover, the intake of high levels of dietary fiber and probiotics has been reported to increase the abundance of SCFA-producing bacteria, induce GLP-1 production, and decrease inflammatory cytokine levels, which is beneficial for the control of various diseases, as mentioned [177]. In addition, the use of antibiotics to treat infectious diseases disrupts the composition of the intestinal microbiota and subsequently stimulates disease progression [190]. Hence, controlled host dietary consumption might serve to regulate intestinal microbiota and their associated metabolites to minimize the risk of disease development.

## 5. Conclusions and Future Perspectives

In this review, we highlighted some examples of microbial metabolites in terms of their production by certain intestinal microbes, their effect on microbial clades and host cells (particularly IECs and immune cells), and their beneficial effects on IBDs and metabolic diseases, including T2D and CVD. Since various microbial metabolites exert similar and common effects, such as enhancing intestinal barrier function, suppressing the pro-inflammatory response, and promoting the anti-inflammatory response, regulation of the intestinal microbiota and related metabolites is important to maintain host health. The production of metabolites by intestinal microbiota has been clearly demonstrated in many studies; however, the effect of such metabolites on microbial clades should be addressed in more detail. Likewise, further investigation is required to determine the degradation or half-life of these metabolites, in addition to how they impact host cells in a dose-dependent manner. In addition, although the interactions between microbial metabolites and associated host diseases have been widely studied, the pathways relevant to the context of specific diseases are only beginning to be addressed. Therefore, further studies to understand the impacts of metabolites on host health and disease are required to drive the modulation of microbial metabolites as a novel approach to improve heath.

## Figures and Tables

**Table 1:** The effect of microbial metabolites on IECs.

Microbial Metabolites	Effect on IECs
SCFAs	Promote epithelial barrier function; Induce IEC proliferation and turnover mediated by activation of MEK–ERK through GPR41 or GPR43; Enhance IEC differentiation and apoptosis; Activate cytokine production via MEK–ERK and p38 MAPK signaling mediated by GPR41 or GPR43; Promote the production of antimicrobial peptides via the activation of mTOR and STAT3 signaling mediated by GPR43; Increase wound healing by stimulating IEC migration through PAKI and MFGE8; Increase the secretion of GLP-1 by L cells.
AHR ligands	Increase tight junction gene expression; Influence IEC differentiation by suppressing enterocyte differentiation and inducing secretory cell differentiation; Decrease pro-inflammatory cytokine production and increase anti-inflammatory cytokine production by attenuating NF-κB.
Bile acids	Regulate IEC integrity by interacting with FXR; Alter colonic cell proliferation and apoptosis depending on concentration; Enhance GLP-1 secretion by L-cells by activating TGR5 and cAMP signaling.
Polyamines	Enhance DNA synthesis; Induce cell migration through Rac1 activation and calcium influx; Promote IEC maturation by increasing glycoprotein galactosylation; Suppress the production of IL-18 and anti-microbial peptides by reducing NLRP6 inflammasome assembly.
Equol	Protect IECs from oxidative damage by promoting the expression of antioxidant genes, enhancing antioxidant enzyme activity; Maintain the integrity of the tight junctions and inhibit IL-8 production; Increase intracellular Ca^2+^ levels, actin reorganization, suppression of GLP-1 secretion by enteroendocrine L cells through GPR30.
Compound K	Enhance SGLT1-mediated glucose uptake by inducing CREB and CBP binding to the *SGLT1* promoter; Inhibit IL-8 secretion by LPS-activated IECs; Inhibit cell growth by arresting the cell cycle in G1 phase; Induce caspase-dependent apoptosis via ROS generation.

IEC, intestinal epithelial cells; SCFAs, short-chain fatty acids; AHR, aryl hydrocarbon receptor; GPR, G-protein coupled receptor; PAK1, p21 activated kinase; MFGE8, milk fat globule-EGF factor 8; GLP-1, glucagon-like peptide-1; NF-κB, nuclear factor kappa-light-chain-enhancer of activated B cells; FXR, farnesoid X receptor; TGR, G-protein coupled receptor; cAMP, cyclic adenosine monophosphate; NLRP6, NOD-like receptor pyrin domain-containing protein 6; SGLT-1, Na(+)/glucose co-transporter 1; LPS, lipopolysacchariade; ROS, reactive oxygen species.

**Table 2:** Effect of microbial metabolites on immune cells.

Microbial Metabolites	Effect on Immune Cells
SCFAs	Inhibit NF-κB-mediated pro-inflammatory cytokine expression; Modulate recruitment of immune cells to the intestinal tract by controlling the expression of chemokines or chemoattractant receptors; Inhibit immune cell proliferation and induce GPR41-dependent cell apoptosis; Reduce the expression of co-stimulatory molecules and induce anti-inflammatory properties in antigen-presenting cells; Contribute to Treg differentiation through increased histone H3 acetylation of *Foxp3* promoter region in a GPR43-dependent manner; Promote homing of Tregs in the colon in a GPR15-dependent manner.
AHR ligands	Inhibit production of pro-inflammatory cytokines such as IL-1β and IL-6 by macrophages; Inhibit migration of macrophages toward chemokines; Induce the expansion and IL-22 production by ILC3; Promote bacterial clearance by macrophages via inducing survival and ROS production; Regulate Treg/Th17 lineage fate by inducing expansion of Tregs, while suppressing Th17 development; Maintain IELs in the intestinal tract.
Bile acids	Block caspase-1 maturation and IL-1 and IL-18 secretion from LPS-primed BMDMs via the TGR5–cAMP–PKA axis; Reprogram pro-inflammatory macrophages to an anti-inflammatory macrophage; Inhibit Th1 activation by binding VDR and inhibiting ERK1/2 phosphorylation.
Polyamines	Inhibit pro-inflammatory cytokine synthesis in LPS-activated monocytes and macrophages; Accelerate intestinal and systemic immune cell maturation, e.g. T cells and B cells.
Equol	Decrease the production of IL-12/IL-18-induced IFN-γ production by natural killer cells; Protect macrophages from LPS-induced oxidative stress by reducing lipid peroxidation and enhancing activity of antioxidant enzymes; Induce antigen-specific IgE production from B cells and IL-13 production from T cells.
Compound K	Inhibit pro-inflammatory cytokine production in LPS-activated macrophages by inhibiting NF-κB; Inhibit macrophage function including polarization and phagocytosis by altering β-arrestin 2 coupling; Induce cell cycle arrest in the G1 phase and apoptosis; Suppress T cell priming by inhibiting the trafficking and signals for T cell activation by dendritic cells.

SCFAs, short-chain fatty acids; AHR, aryl hydrocarbon receptor; NF-κB, nuclear factor kappa-light-chain-enhancer of activated B cells; GPR, G-protein coupled receptor; Tregs, regulatory T cells; ILC3, group 3 of innate lymphoid cell; ROS, reactive oxygen species; Th, helper T cell; VDR, vitamin D receptor; LPS, lipopolysaccharide.

## Abbreviations

AHR	aryl hydrocarbon receptor
BMDM	bone marrow-derived macrophage
cAMP	cyclic adenosine monophosphate
CBP	CREB-binding protein
CNS	central nervous system
CREB	cAMP response element binding protein
CVD	cardiovascular disease
DC	dendritic cell
DCA	deoxycholic acid
ERK	extracellular signal-regulated kinases
FXR	farnesoid X receptor
GI	gastrointestinal
GLP-1	glucagon-like peptide-1
GPR	G-protein coupled receptor
HDAC	histone deacetylase
I3C	indole-3-carbinol
IBD	inflammatory bowel disease
IEC	intestinal epithelial cell
IEL	intraepithelial lymphocyte
IL	interleukin
ILC	innate lymphoid cell
ILC3	group 3 of innate lymphoid cell
LCA	lithocholic acid
LPS	lipopolysaccharide
MAPK	mitogen-activated protein kinase
MEK	mitogen-activated protein kinase
MFGE8	milk fat globule-EGF factor 8
mTOR	mammalian target of rapamycin
NAFLD	non-alcoholic fatty liver disease
NF-κB	nuclear factor kappa-light-chain-enhancer of activated B cells
NLRP	NOD-like receptor pyrin domain-containing protein
PAKI	p21 activated kinase
ROS	reactive oxygen species
SCFA	short-chain fatty acid
SGLT1	Na(+)/glucose co-transporter 1
STAT	signal transducer and activator of transcription
T2D	type 2 diabetes
TGR	G-protein-coupled receptor
Th	helper T cell
TNF	tumor necrosis factor
Treg	regulatory T cell
VDR	vitamin D receptor
